# The Effect of p-Toluenesulfonic Acid and Phosphoric Acid (V) Content on the Heat Resistance and Thermal Properties of Phenol Resin and Phenol-Carbon Composite

**DOI:** 10.3390/ma17235914

**Published:** 2024-12-03

**Authors:** Łukasz Rybakiewicz, Janusz Zmywaczyk

**Affiliations:** 1 Military Institute of Armament Technology, Prymasa Stefana Wyszyńskiego 7 Str., 05-220 Zielonka, Poland; 2 Faculty of Mechatronics, Armaments and Aerospace of Military University of Technology, Kaliskiego 2 Str., 00-908 Warsaw, Poland

**Keywords:** phenolic resin, thermal stability, thermophysical parameters

## Abstract

This work presents the results of research on the influence of the amount of p-toluenesulfonic acid and phosphoric acid (V) added to the phenol-formaldehyde resin (pH 7.3–7.8) on its thermal properties and on the phenol-formaldehyde-carbon composite produced on its basis. This material undergoes pyrolysis under high temperature. The addition of a catalyst to the phenol-formaldehyde resin affects its curing rate and degree of cross-linking, but how it affects the thermal properties of the resin depending on the temperature is the subject of this work. This article presents the results of thermal tests for phenol-formaldehyde resin and phenol-formaldehyde-carbon composite. It was examined how the content of the catalyst used during the production process affects the individual thermal parameters of the mentioned materials. The results include experimental tests of thermal diffusivity with uncertainty (±3%), specific heat capacity (±2.5%), thermal expansion with resolution 2 nm analyzed in the temperature range −40–115 °C and thermogravimetric TG/DTA analysis with resolution 0.03 µg in the temperature range from room temperature (*RT* = 23 °C) to 550 °C. Individual thermal tests showed changes in the thermal properties caused by changes in the catalyst content of the tested materials and the influence of the addition of carbon fibers on the properties of the composite compared to the pure phenol-formaldehyde resin. It was found that there is a certain maximum level of catalyst weight fraction at which the greatest decrease in thermal diffusivity occurs. In the case of phenolic-formaldehyde-carbon composite at −40 °C, an increase in catalyst weight fraction from 2 to 4 wt% caused a decrease in thermal diffusivity by 18.2%, and for phenol-formaldehyde resin, it was 2.8% with an increase in catalyst fraction from 4 to 10 wt%.

## 1. Introduction

Phenolic resins are synthetic thermosetting polymers that are characterized by low flammability, formation of a carbon layer on the surface when exposed to a high-density heat stream and chemical stability. Moreover, these resins are very good and stable insulators [1,2]. Phenolic resins are most often used for the production of heat shields for thermally sensitive parts of machines, devices that are exposed to high temperatures or for the production of structural elements made of phenolic-formaldehyde-carbon composite (PFCC). Combined with carbon fibers, they constitute a material that is used in the space and arms industries. It is characterized by high strength and very good thermal properties.

The properties of the fibers, i.e., their arrangement, diameter and thermal conductivity, influence the thermal properties of the composite, especially when it is used to build ablative thermal shields [3]. To increase its thermal resistance, a number of chemical admixtures or additives can be used that influence the ablation rate. In the case of the addition of zirconium diboride, compounds with zirconium are formed during the transformation, which protect the surface of the ablative material from the effects of high temperature. The addition of carbon nanotubes or graphene oxide also has a positive effect on the ablation properties [4]. Composite production is very complex and is influenced by a number of factors to obtain a repeatable product. Curing and polymerization are the most critical during its production. Appropriate regulation of parameters such as pressure and temperature are key to achieving high-quality composites [5]. In the production of PFCC, a chemical hardener is used, whose task is to fix the shape of the manufactured part and finally harden it at an elevated temperature [4]. The structure of phenol resin is diverse, which contributes to the complex curing process. In different types of phenolic resins, the course of the hardening reaction is also different [6]. Composite optimization can take place at various production stages. One of them is the selection of a chemical hardener in the case of phenolic resins, after which the resin is initially hardened within a specified time. Phenol resins can be pre-hardened by cross-linking monomers, initiators or catalysts, which differ from initiators in that they are not incorporated into the structure of the cross-linked polymer but are regenerated in the finished product [7]. The effect of the hardener content in phenol-formaldehyde resin (PFR) on its thermal properties is an often overlooked topic and there are few studies on this effect. The work [8] presents how a basic catalyst of formaldehyde and phenol reactions affect the kinetics and mechanisms of addition, as well as condensation mechanisms. The paper presents the influence in chemical terms. The work [9] also presents the chemical effect of trimethylamine catalysts. The amount of hardener added influences the condition and properties of the resin in the production process, i.e., how long it will be in a liquid state.

The authors of this paper drew attention to the problem of how the percentage of catalyst added during production affects the thermal properties of PFR as well as PFCC composite. Increasing the amount of catalyst reduces thermal diffusivity and increases relative elongation while shortening the resin cure time. The observed effect can be used to determine the optimal catalyst addition at which thermal diffusivity in a given temperature range will be the highest, which has a beneficial effect on the rate of temperature equalization in the unsteady state. A high-quality composite filled with phenolic resin is a good material to protect structures against destructive heat transfer into their structure [3,10]. Composites based on phenolic resins are used to produce protective coatings for nozzles, combustion chambers, and rocket parts that are exposed to high temperatures during their entry into the atmosphere. They are also used as an ablative material in rocket engines [11,12,13]. The literature review shows that the unresolved problem is to determine how the thermal diffusivity, thermal expansion and effective specific heat of the phenol-formaldehyde resin changes quantitatively depending on the weight fraction of the p-toluenesulfonic acid and phosphoric acid catalyst.

The aim of this work is to experimentally determine the effect of the weight fraction of p-toluenesulfonic acid and phosphoric acid (V) selected for precuring on the thermal properties of pure phenol-formaldehyde resin and phenol-formaldehyde-carbon composite. This work explains how the catalyst affects the thermal properties and heat resistance of PFR and PFCC and gives an answer to whether the thermal properties can be improved by changing the amount of catalyst. The obtained experimental results can be used for numerical modeling of heat transfer processes in phenol-formaldehyde composites reinforced with carbon fibers.

## 2. Experimental Program

Samples of composites based on phenol-formaldehyde resin were tested for thermal properties. For this purpose, specialized measurement equipment purchased from the German NETZSCH leading company (NETZSCH Holding—Group, Selb, Germany) was used, in the form of a thermogravimetric analyzer STA 2500 Regulus, a low-temperature dilatometer DIL 402 Expedis, a DSC 404 F1 Pegasus microcalorimeter and a light flash analyzer LFA 467 HyperFlash.

*Materials.* Phenol-formaldehyde resin with a catalyst acid added during production for initial hardening of the resin, provided by LERG S.A (Pustków, Poland), was used for the tests. The resin is a mixture: phenol, polymer with formaldehyde (CAS: 9003-35-4), phenol (CAS: 108-95-2) and formaldehyde (CAS: 50-00-0). The catalyst is a mixture: p-toluenesulfonic acid (CAS: 104-15-4) and phosphoric (V) acid (CAS: 7664-38-2). Physicochemical properties of the resin: viscosity at 20 °C–700–800 mPas, non-volatile matter content 135 °C/3 g/1 h-70–74%, pH 7.3–7.8, free formaldehyde content < 3%, free phenol content < 10 %. Catalyst: light brown liquid, density at 20 °C, 1.2–1.3 g/cm^3^. Carbon fibers were purchased from Toray Industries, Inc. They were used in the form of fabrics in a 2/2 twill arrangement. The addition of carbon fibers to the PFR was intended to reduce the linear erosion rate of the composite, increase its mechanical strength and control heat flow through the composite.

The test samples were made with different catalyst contents (%wt) 4%, 7% and 10% (only resin samples), respectively. Composite samples with a catalyst content of 10% were not made because the resin was liquid for too short a time. Phenol-formaldehyde-carbon composite (PFCC) was made by infusion. The names and purpose of all samples selected for testing are given in Table 1. The percentage content of resin to fiber was 45% to 55%, respectively. This proportion results from the possibilities that are obtained by increasing the fiber content using the infusion method in the production process [14]. PFR samples and PFCC samples were heated at 60 °C for 3 h and at 80 °C for 6 h to cure.

**Figure 1:**
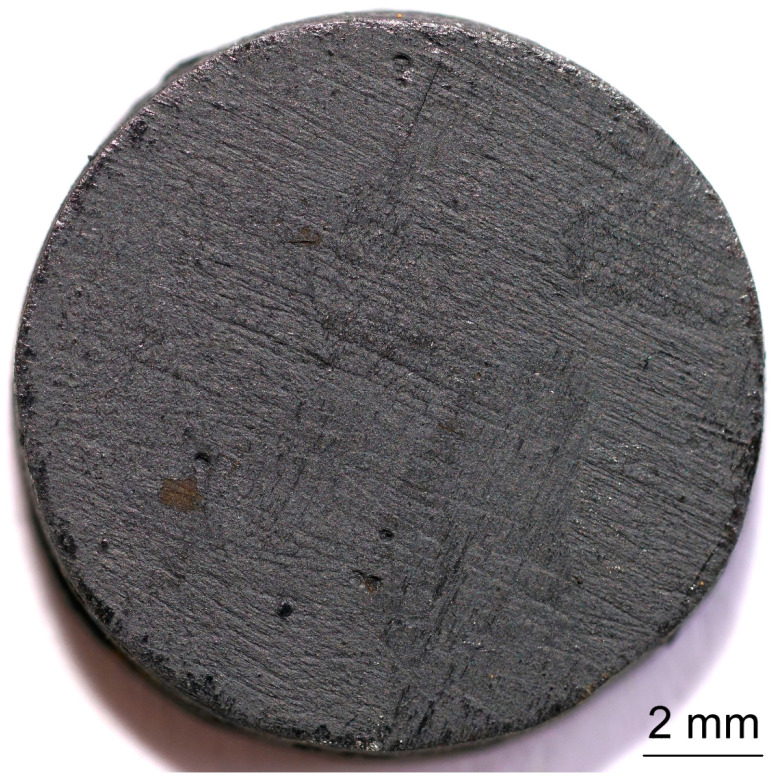
A sample of PFCC for thermal diffusivity testing using the pulse method with a graphite layer several micrometers thick.

**Figure 2:**
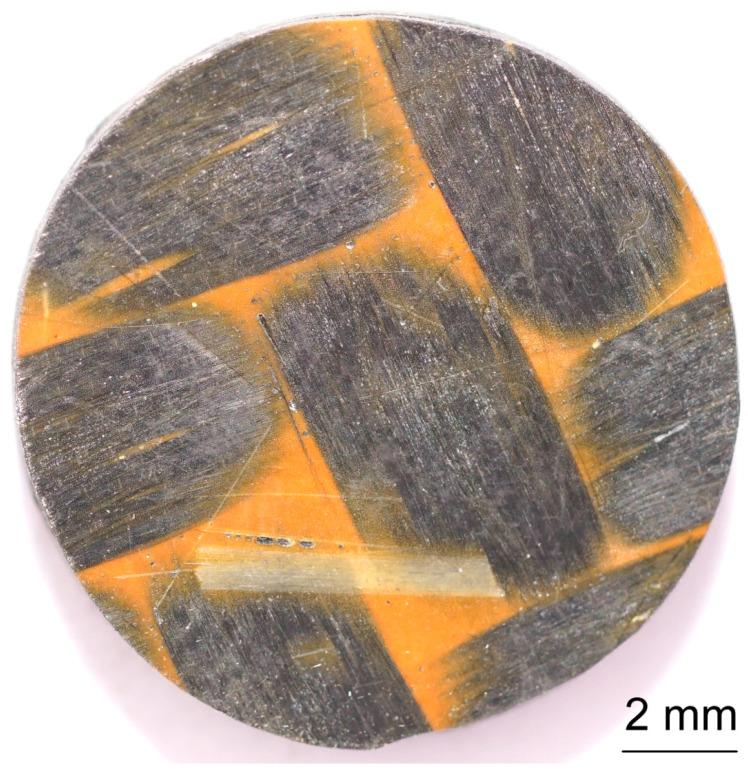
A sample of PFCC for thermal diffusivity testing using the pulse method without a graphite layer several micrometers thick.

The samples for thermal diffusivity testing had the shape of a cylinder with a diameter of about ø12.6 mm and a thickness of about 2.1 mm, while the samples for thermal expansion testing using the dilatometric method had a diameter of about ø5.5 mm and a length of about 15.5 mm. Samples with dimensions of ø12.6 mm × 2 mm were made of PFR and PFCC with fibers arranged across the diameter. Samples for thermal diffusivity tests are presented in Figure 1 and Figure 2. Samples with dimensions of ø5.5 mm × 15.5 mm were made of PFCC with fibers arranged along and across the diameter. Shafts were cut out from previously prepared plates in two directions. The densities of the test samples were determined using the buoyancy method (weighing in air and water) using a Sartorius Cubis II lab balance.

TG/DTG/DTA (Thermogravimetry/Derivative Thermogravimetry/Differential Thermal Analysis) tests were performed on a small section of the composite weighing approximately 4 mg. The tests were conducted in a helium environment—50 mL/min at a heating rate *HR* = 10 K/min in the temperature range 30–550 °C on the sample PFCC-4C_Fibers using STA 2500 Regulus.

Samples were used for testing the thermal diffusivity: PFR-4C, PFR-7C, PFR-10C, PFCC-2C, PFCC-4C. The thermal diffusivity studies were performed using LFA 467 HyperFlash in an argon environment of 50 mL/min in the temperature range −40–140 °C. Liquid nitrogen was used to lower the temperature of the measuring chamber below zero. The xenon lamp parameters were as follows: lamp voltage 250 V, pulse width 600 µs. For example, for the PFR-4C sample, the duration of 6000 ms and the gain amplitude of 10,000 × 145 were set at a temperature of 20 °C. The front and back surfaces of the thermal diffusivity test specimen were sprayed with a conductive coating of Graphit 33 (CRC Industries Europe BV, 9240 Zele, Belgium) with a thickness of several micrometers. Samples for thermal diffusivity are shown in Figure 1 and Figure 2.

**Figure 3:**
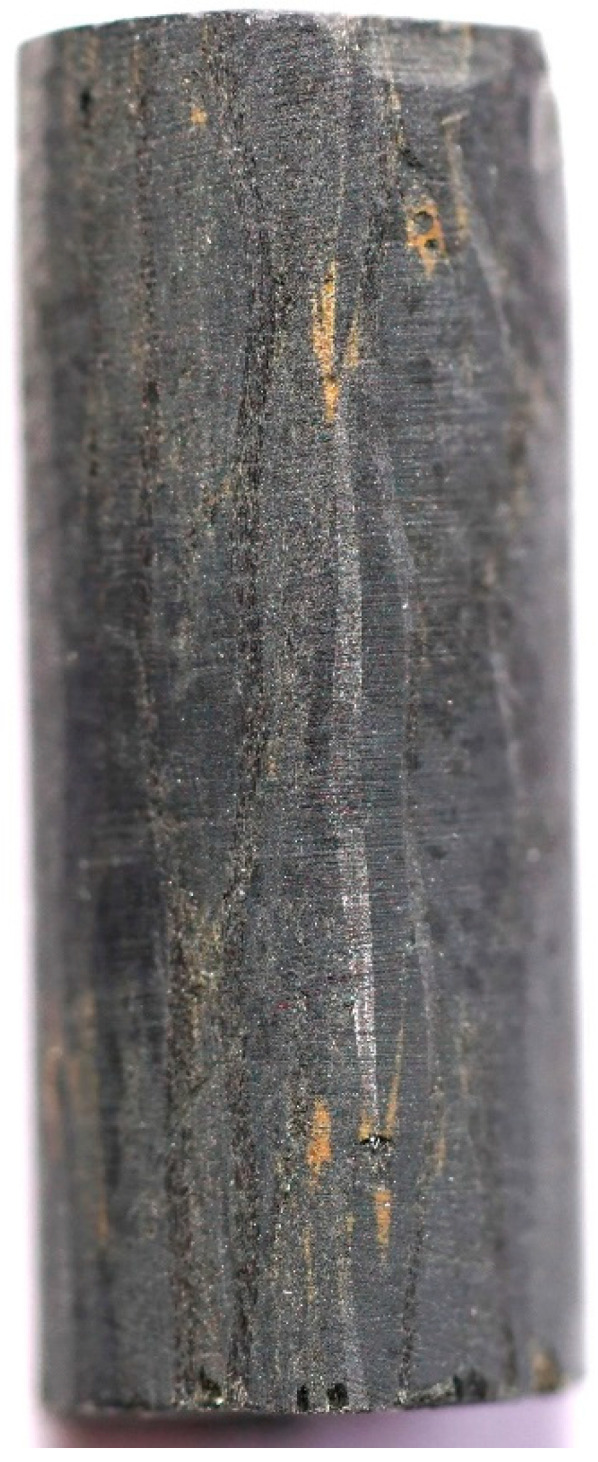
A sample of PFCC for thermal expansion tests cut along the fibers.

**Figure 4:**
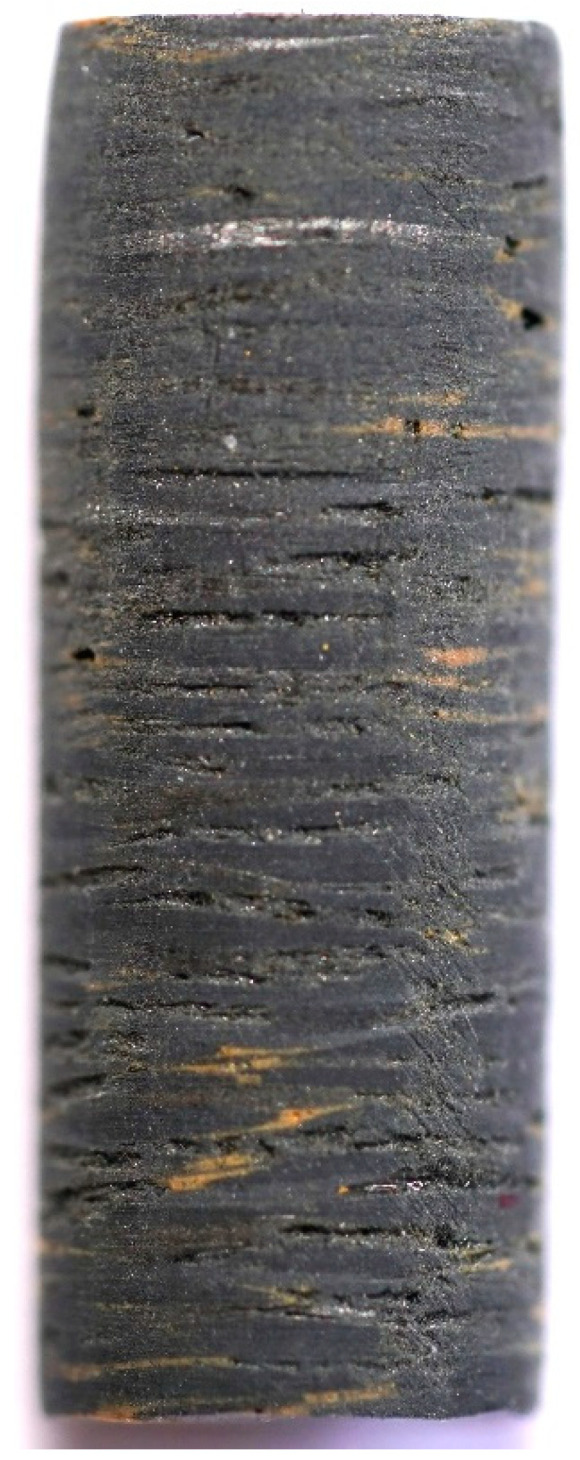
A sample of PFCC for thermal expansion tests cut perpendicular to the fibers.

Samples PFCC-2C-AL, PFCC-2C-AC, PFCC-4C-AL and PFCC-4C-AC were used for thermal expansion tests. Figure 3 shows a view of the sample with carbon fibers arranged along the sample axis with a length of 15.5 mm, while Figure 4 shows carbon fibers as fabric arranged transversely to the sample axis. The tests were carried out using a low-temperature dilatometer DIL 402 Expedis while maintaining a constant heating and cooling rate of 2 K/min in the temperature range from −50 to 170 °C in an argon environment of 20 mL/min.

**Figure 5:**
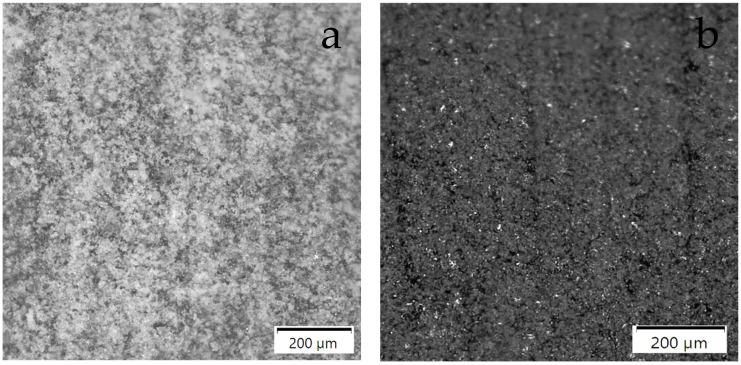

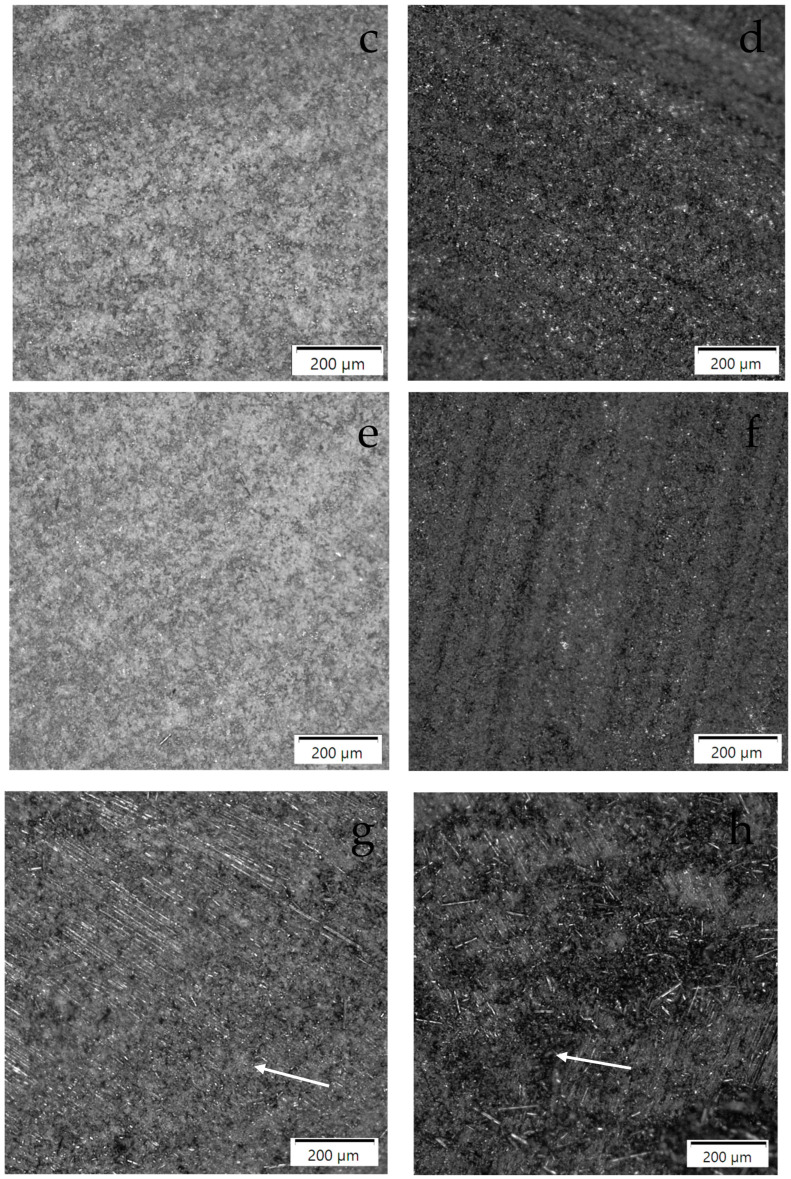

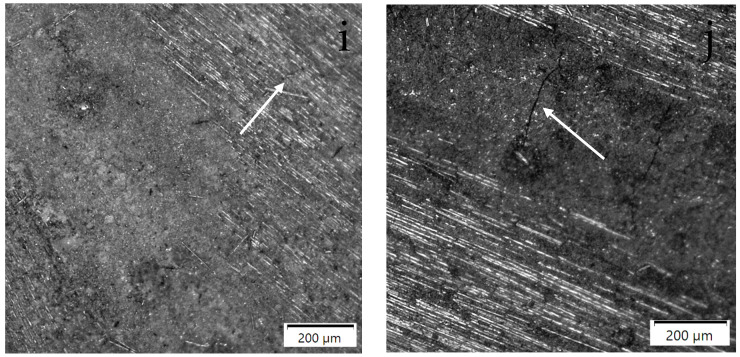
Photos of PFR and PFCC: (**a**) PFR resin sample with 4% wt catalyst before testing, (**b**) PFR sample with 4% wt catalyst after testing at 370 °C, (**c**) PFR sample with 7% wt catalyst before testing, (**d**) PFR sample with 7% wt catalyst after testing at 370 °C, (**e**) PFR sample with 10% wt catalyst before testing, (**f**) PFR sample with 10% wt catalyst after testing at 370 °C, (**g**) PFCC sample with 2% wt catalyst in the resin before testing, (**h**) PFCC sample with 2% wt catalyst in the resin after testing, (**i**) PFCC sample with 4% wt catalyst in the resin before testing, (**j**) PFCC sample with 4% wt catalyst in the resin after testing.

The apparent specific heat tests were carried out using PFR-4C, PFR-7C, PFR-10C, PFCC-2C and PFCC-4C samples with dimensions of diameter × thickness, approximately 5.3 mm × 1.5 mm. The DSC tests were conducted in an argon environment of 50 mL/min at HR = 5 K/min in the temperature range of 23–380 °C using DSC 404 F1 Pegasus. Below are enlarged photos of selected samples. In enlarged photos, it is possible to observe the distribution of the resin and carbon fibers in the composite (Arrows: Figure 5g—resin, Figure 5h—carbonized resin, Figure 5i—fibers), as well as the course of the fibers in the case of composites. By observing the samples under magnification, it is possible to detect surface heterogeneities of the material. The resin structure with higher catalyst content is finer (Figure 5a,c,e). Figure 5b,d,f present resin after testing at a temperature of 370 °C. Samples (Figure 5h,j) represent the results of resin pyrolysis, where resin degrades and carbon fibers are exposed. There is a noticeable crack (indicated by an arrow) in Figure 5j. PFCC structure before testing is presented in Figure 5g,i.

## 3. Results and Discussions

**Figure 6:**
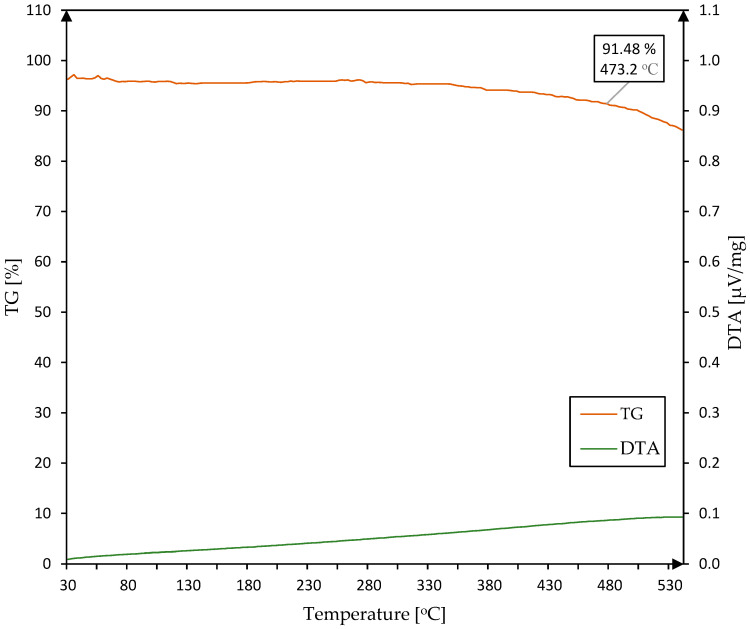
TG and DTA temperature characteristics of the PFCC with 4% wt catalyst in resin content.

The TG/DTA thermogram (Figure 6) presents the process of thermal degradation of the PFCC containing 4% of a catalyst in resin during its continuous heating at a rate of 10 °C/min in the range from 30 °C to 540 °C. The beginning of the chart begins from 96.3% which due to incorrect balancing does not allow determining 100% of the sample mass before testing. It has not influenced the result. A 5% decrease (91.48% on the chart) in the initial sample mass was observed after reaching a temperature of 473.2 °C. In the range from 30 °C to 473.2 °C, the derivative thermogravimetry curve was almost horizontal, which means that the thermal degradation rate is constant or does not occur. A sharp increase in thermal degradation is observed when the temperature exceeds 538 °C, where the next stage of degradation begins [15]. At the end of the heating process, when the temperature reached 540 °C, 86.2% of the initial mass of the sample remained and its surface became charred. Structural changes for phenolic-formaldehyde resin during pyrolysis processes have been described in the work [16]. TG/DTA tests for higher temperatures were not performed due to the melting temperature (660.3 °C) of the aluminium crucibles used.

**Figure 7:**
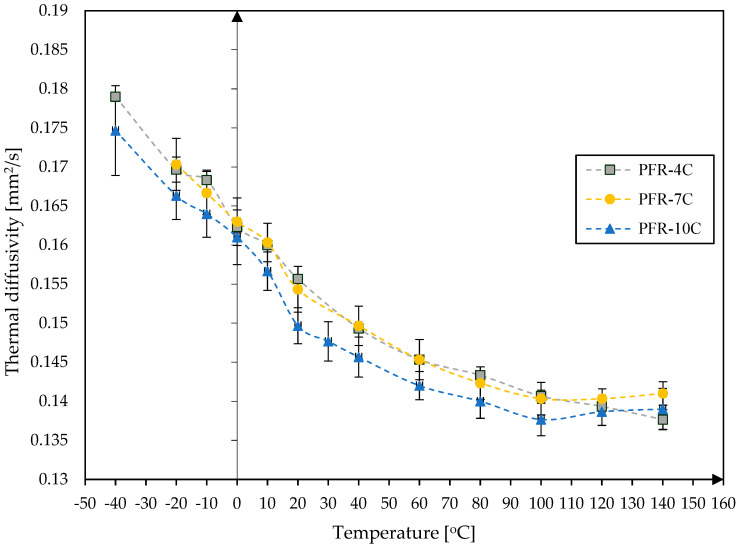
Thermal diffusivity depending on temperature for phenolic resins with 4%, 7%, 10% wt catalyst content.

**Figure 8:**
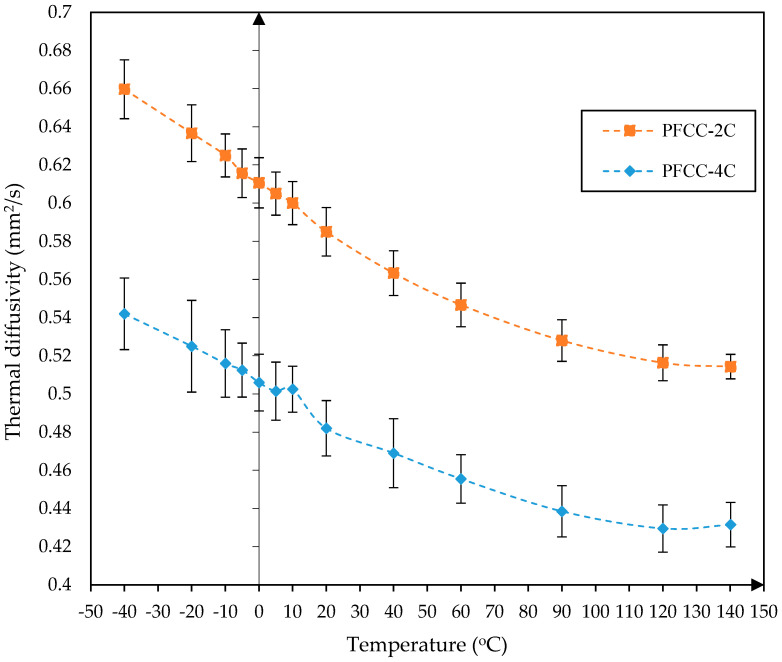
Thermal diffusivity depending on temperature for PFCC with 2% and 4% wt catalyst in resin content.

Since the thermal diffusivity tests used a sample made of the reference material Pyroceram 9606 from NETZSCH, it was possible to additionally determine the volumetric heat capacity and thermal conductivity of the tested samples of PFR and PFCC. Their thermal diffusivity temperature characteristics obtained by fitting the standard model (Cape–Lehman) with pulse correction to the measured thermal response signal on the back surface of the sample using an IR sensor are shown in Figure 7 and Figure 8.

It should be noted that during the thermal diffusivity tests, the assumption was made that the sample mass was constant. An increase in thermal diffusivity with an increase in the catalyst content was observed in the case of the PFCC, while in the case of the resin, the thermal diffusivity is comparable for all samples; the difference is less than 5%. For samples PFR-4C and PFR-7C, the results are mostly similar. Comparison of the results of the tests of the catalyst effect on the thermal diffusivity of phenolic resin and PFCC leads to the conclusion that the increase in catalyst content causes a greater variability of the thermal diffusivity of PFCC (about 20%) than that of PFR (about 5%). The addition of carbon fibers increases the thermal diffusivity several times. In both cases, catalyst content influences thermal diffusivity. Composites are widely used compared to pure resin, so catalyst content matters more. For PFCC, the thermal diffusivity increases on average from 0.42 mm^2^/s to 0.66 mm^2^/s, while for samples made of PFR, the thermal diffusivity is several times lower and ranges from 0.138 to 0.179 mm^2^/s. Thermal diffusivity reaches its highest value at −40 °C, which is 0.66 mm^2^/s for PFCC-2C. The minimum value of thermal diffusivity occurs at 100 °C for PFR-10C, at 140 °C for PFR-4C and amounts to 0.138 mm^2^/s.

The results of the thermal diffusivity of phenol-formaldehyde resin dopped with p-toluenesulfonic acid and phosphoric acid (V) in the ratio given by Motram and Taylor for phenolic resin SC-1008 showed agreement in terms of the order of magnitude. It is difficult to compare the results of the thermal diffusivity with each other because the measurement ranges are different. In addition, around 0 °C there may be a decrease in thermal diffusivity, which was observed during laboratory tests. This is probably related to water freezing. This is most likely also related to the production process or the storage time of the samples and storage conditions.

**Figure 9:**
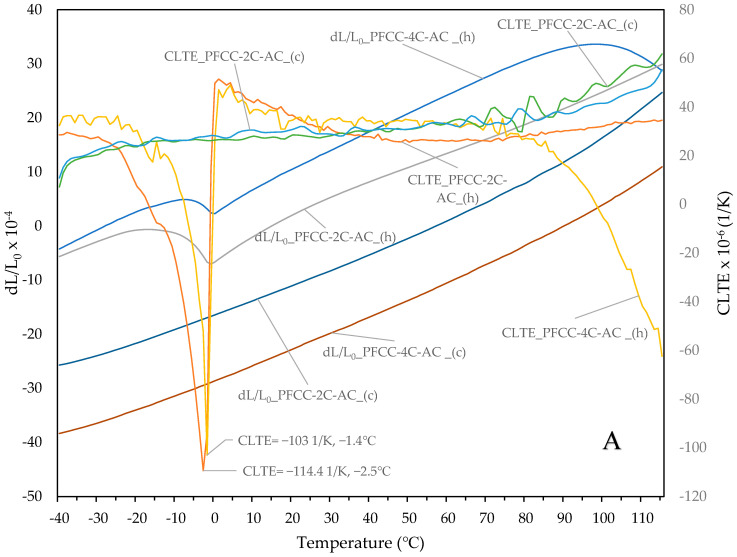

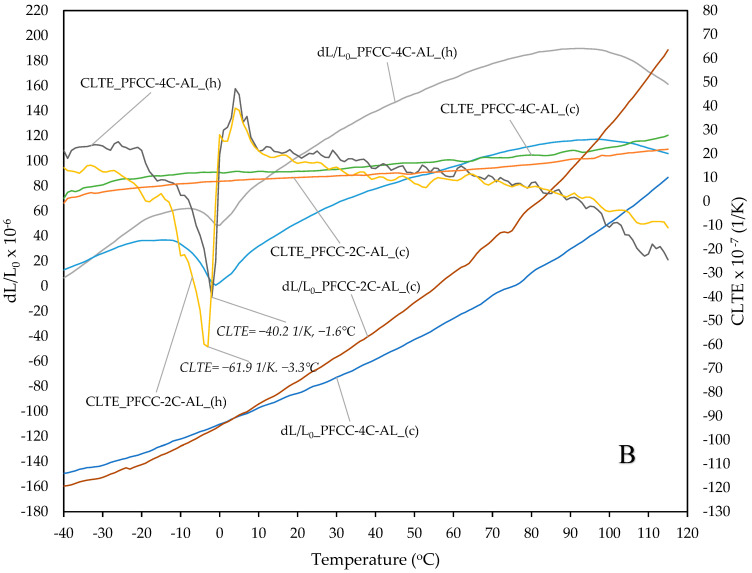
Relative elongation (dL/L_0_) and coefficient of linear thermal expansion (CLTE) of a PFCC with carbon fibers arranged in the following directions: (**A**) transverse (PW) and (**B**) along the length of the sample (WW); (h) stands for heating, (c) for cooling.

The temperature characteristics of relative elongation dL/L_0_ and coefficient of linear thermal expansion (CLTE or denoted by NETZSCH as diff-CLTE) of the tested samples are shown in Figure 9A,B. Comparing the CLTE test results of the PFCC-2C and PFCC-4C composites shown in Figure 9A,B, it can be seen that the material shrinkage around −2 °C during heating is almost twice as high for the fibers arranged across the sample compared to fibres arranged along the sample and decreases with the increasing catalyst content. The jagged curve of the coefficient of linear thermal expansion (CLTE) function results from the superposition of noise on the measurement signal. Since the CLTE is calculated as the first derivative of the relative sample elongation dL/L_0_ with respect to temperature, the numerical differentiation operation leads to the amplification of the disturbance amplitude. The amplitude of the noise can be mitigated by a chosen measurement error smoothing technique, but this may lead to the loss of some information. Analysis of the behavior of CLTE allows to conclude that as the temperature increases, thermal shrinkage of the material occurs, starting at the onset temperature −12 °C for PFCC-2C-AC (peak −2.5 °C CLTE = −114.4 × 10^−6^ K^−1^) and at −9.4 °C for PFCC-4C-AC (peak −1.4 °C CLTE = −103·10^−6^ K^−1^). In the temperature range from 40 to 115 °C, the CLTE is virtually constant with a value of about 26·10^−6^ K^−1^ for the PFCC-2C-AC composite. In the case of the PFCC-4C-AC, the CLTE has a practically constant value equal to 34·10^−6^ K^−1^ in the range of 20 to 70 °C, and then decreases quite rapidly from 34·10^−6^ K^−1^ to −59·10^−6^ K^−1^ (Figure 9A). The coefficient behaves similarly for composites with fibers arranged along the length of the specimen. Thermal shrinkage begins at the onset temperature of the −12.1 °C for PFCC-2C-AL (peak −3.3 °C CLTE = −6.19·10^−6^ K^−1^) and at −10.1 °C for PFCC-4C-AL (peak −1,6 °C CLTE = −4.02·10^−6^ K^−1^). In the temperature range of 10 to 115 °C CLTE for both types of composites, there were slight decreases in the range from 2.19 ·10^−6^ K^−1^ to −0.67 ·10^−6^ K^−1^ for PFCC-2C-AL and −2.10 ·10^−6^ K^−1^ to −2.58 ·10^−6^ K^−1^ for PFCC-4C-AL (Figure 9B).

**Figure 10:**
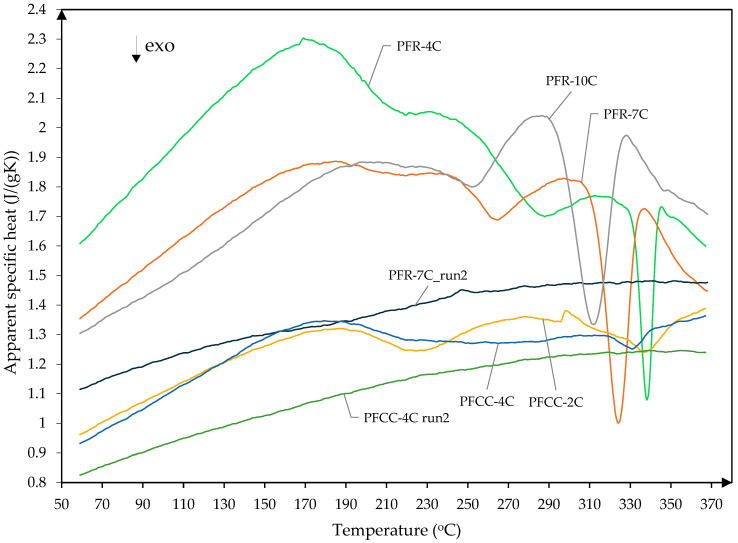
Apparent specific heat versus temperature for PFR with 4%, 7% and 10% wt catalyst and PFCC with 2% and 4% wt catalyst (exo—downwards).

Apparent specific heat depending on temperature is shown in Figure 10 for PFR and PFCC. Every sample showed a rapid decrease in specific heat in the temperature range of 290 °C to 350 °C. Decreases in apparent specific heat were observed for both the composite and the resin. This decline is related to the exothermic reaction. After cooling the sample and repeating the test, the decrease did not occur for PFCC. Considering that the samples were curing at 80 °C, this decrease is associated with structural changes. As a result of these changes, an exothermic reaction occurred. This transformation may be related to the curing of the resin as it did not occur after cooling the sample and repeating the test. The article [17] presents the course of the DSC signal during resin curing. During hardening, endothermic and exothermic reactions occur several times. If the hardening took place at a high temperature, e.g., 300 °C, a significant part of the resin would decompose, which was confirmed in work [17].

Resin samples showed higher apparent specific heat compared to PFCC. In addition, there was a shift in the exothermic reactions depending on catalyst content. This phenomenon occurs both for the resin and composite. The first endothermic peak of specific heat occurs at 172 °C for PFR-4C resin and at 176 °C for PFCC-4C (Figure 10). Based on the results of Zhang et al. [17], it can be assumed that at a temperature of 172 °C, the evaporation of water and phenol molecules occurs. The TG/DTA tests (Figure 6) show that the mass loss is small and amounts to 1%. At 331.4 °C for PFCC-4C and at 338.3 °C for PFR-4C, exothermic peaks occur, which are probably the effect of dehydration–condensation reactions. During the exothermic reaction, the decrease in apparent specific heat is much greater for phenolic resin (−38%) than for composite (−4%). The observed experimental facts are the result of the course of polymerization processes during the production of the tested phenolic resins. Due to the lack of complete information on the phenolic resin in the works [17,18], it is difficult to directly compare the results of our own studies with those of other phenolic resins, such as rezol (5408) phenolic resin [17] or SC-1008 [18].

Table 2 shows the onset, end, peak temperatures and enthalpies of exothermic phase transformations visible in Figure 10 in the temperature range of 290–350 °C determined from DSC thermograms. The peak tendency and the amount of energy released to the environment are immediately visible. In the case of the PFR-4C sample, the peak occurred at a temperature of 343.3 °C, while for the PFR-10C sample at a temperature of 323.6 °C. An analogous situation occurs for the PFCC. The peak for the PFCC-2C sample occurred at a temperature of 349.4 °C, while for the PFCC-4C sample at a temperature of 331.4 °C. The energy released to the environment increases with the increase in the amount of added catalyst.

The amount of catalyst added influences the structure of the phenol-formaldehyde resin and thus its thermal properties. Thermal expansion tests have shown that the weight fraction of the catalyst in the carbon composite (Figure 9) has a significant effect on the relative elongation dL/Lo of the PFCC-2C and PFCC-4C. For example, at a temperature of 20 °C in the direction of the carbon fibers along the sample axis during heating, the relative elongation of the PFCC-4C is two times greater than that of the PFCC-2C and reduction of about 20% greater during cooling (Figure 9B), and in the case of transversely arranged fibers, this change is two times during heating and two times during cooling (Figure 9A). Comparing the change in the relative elongation of composites with fibers along and across the sample axis during heating, it is 27 times greater and 10 times greater during cooling. Thermal diffusivity tests showed about a 20% increase in its value for the PFCC-2C compared to PFCC-4C (Figure 8) and about a 5% decrease for the phenolic resin with 10% catalyst content compared to the resin with 4% catalyst content (Figure 7).

## 4. Conclusions

1. The test results confirmed that the influence of the content of the catalyst initiating the curing of the PFR adding during production affects its thermal properties similar in the case of PFR and in the case of PFCC. The catalyst is incorporated into the resin structure, which is confirmed by apparent specific heat tests. This affects the structure of the resin. In the case of a composite, the resin is close to 50% of the material, so this is a significant amount that affects the properties of the PFCC.
2. In the case of the PFCC used as an insulator, it is better to use the resin with a higher catalyst content because its thermal diffusivity is lower and apparent specific heat is higher. It is analogous in the case of PFR. Regarding thermal expansion, it is better to have a PFCC with a lower catalyst content, because it has greater geometric stability.

## Figures and Tables

**Table 1:** List of tested samples. STA—thermogravimetry, LFA—thermal diffusivity, DIL—thermal expansion.

Name	Description	Approximate Dimensions	Density g/cm^3^	Type of Examination
PFCC-4C	Phenol-formaldehyde-carbon sample—4% catalyst	-		STA
PFR-4C	Phenolic sample—4% catalyst	ø12.7 × 2.3 mm	1.01	LFA
PFR-7C	Phenolic sample—7% catalyst	ø12.5 × 2.1 mm	1.01	LFA
PFR-10C	Phenolic sample—10% catalyst	ø12.5 × 2.1 mm	0.99	LFA
PFCC-2C	Phenol-formaldehyde-carbon sample—2% catalyst in resin	ø12.9 × 2.1 mm	1.49	LFA
PFCC-4C	Phenol-formaldehyde-carbon sample—4% catalyst in resin	ø12.8 × 2.1 mm	1.40	LFA
PFCC-2C-AL	Phenol-formaldehyde-carbon fiber sample along the dimension of 15 mm—2% of the catalyst in resin	ø 5.3 × 15.4 mm	1.51	DIL
PFCC-2C-AC	Phenol-formaldehyde-carbon fiber sample across 15 mm dimension—2% catalyst in resin	ø 5.7 × 15.5 mm	1.50	DIL
PFCC-4C-AL	Phenol-formaldehyde-carbon fiber sample along the dimension of 15 mm—4% of the catalyst in resin	ø 5.0 × 15.4 mm	1.51	DIL
PFCC-4C-AC	Phenol-formaldehyde-carbon fiber sample across 15 mm dimension—4% catalyst in resin	ø 5.7 × 15.6 mm	1.50	DIL

**Table 2:** The DSC analysis values for the tested materials whose apparent specific heat are shown in Figure 10.

Resin	Onset (°C)	End (°C)	Peak (°C)	Enthalpy (J/g)
PFR-4C	331.9	343.3	338.3	−4.70
PFR-7C	312.0	333.1	324.2	−9.15
PFR-10C	293.5	323.6	312.1	−11.74
PFCC-2C	328.6	349.4	338.0	−0.84
PFCC-4C	318.3	340.0	331.4	−0.73

## Data Availability

The data presented in this study are available on request from the corresponding author due to privacy.
